# The *Plasmodium falciparum* chloroquine resistance transporter is associated with the ex vivo *P. falciparum* African parasite response to pyronaridine

**DOI:** 10.1186/s13071-016-1358-z

**Published:** 2016-02-09

**Authors:** Marylin Madamet, Sébastien Briolant, Rémy Amalvict, Nicolas Benoit, Housem Bouchiba, Julien Cren, Bruno Pradines, the French National Centre for Imported Malaria Study Group

**Affiliations:** Equipe Résidente de Recherche en Infectiologie Tropicale, Institut de Recherche Biomédicale des Armées, Hôpital d’Instruction des Armées Laveran, Marseille, France; Unité de Recherche sur les Maladies Infectieuses et Tropicales Emergentes, Aix Marseille Université, UM 63, CNRS 7278, IRD 198, Inserm 1095, Marseille, France; Centre National de Référence du Paludisme, Marseille, France; Unité de Parasitologie et d’Entomologie, Département des Maladies Infectieuses, Institut de Recherche Biomédicale des Armées, Brétigny sur Orge, France; Direction Interarmées du Service de Santé, Cayenne, Guyane France; Laboratoire de Parasitologie, Institut Pasteur de la Guyane, Cayenne, Guyane France

**Keywords:** Malaria, *Plasmodium falciparum*, Antimalarial, Resistance, In vitro, Molecular marker, *pfcrt*

## Abstract

**Background:**

The pyronaridine-artesunate combination is one of the most recent oral artemisinin-based therapeutic combinations (ACTs) recommended for the treatment of uncomplicated *P. falciparum* malaria. The emergence of *P. falciparum* resistance to artemisinin has recently developed in Southeast Asia. Little data are available on the association between pyronaridine susceptibility and polymorphisms in genes involved in antimalarial drug resistance. The objective of the present study was to investigate the association between *ex vivo* responses to pyronaridine and the K76T mutation in the *pfcrt* gene in *P. falciparum* isolates.

**Methods:**

The assessment of *ex vivo* susceptibility to pyronaridine was performed on 296 *P. falciparum* isolates using a standard 42-h 3H-hypoxanthine uptake inhibition method. The K76T mutation was also investigated.

**Results:**

The pyronaridine IC_50_ (inhibitory concentration 50 %) ranged from 0.55 to 80.0 nM. *Ex vivo* responses to pyronaridine were significantly associated with the K76T mutation (*p*-value = 0.020). The reduced susceptibility to pyronaridine, defined as IC_50_ > 60 nM, was significantly associated with the K76T mutation (*p*-value = 0.004). Using a Bayesian mixture modelling approach, the pyronaridine IC_50_ were classified into three components: component A (IC_50_ median 15.9 nM), component B (IC_50_ median 34.2 nM) and component C (IC_50_ median 63.3 nM). The K76T mutation was represented in 46.3 % of the isolates in component A, 47.2 % of the isolates in component B and 73.3 % of the isolates in component C (*p*-value = 0.021).

**Conclusion:**

These results showed the *ex vivo* reduced susceptibility to pyronaridine, i.e., IC_50_ > 60 nM, associated with the K76T mutation.

In 2002, the World Health Organization (WHO) recommended the use of artemisinin-based combination therapy (ACT) for the treatment of all cases of uncomplicated malaria. The pyronaridine-artesunate combination (Pyramax®) is one of the latest oral ACTs recommended for the treatment of uncomplicated *P. falciparum* and *P. vivax* malaria [1]. The combination pyronaridine-artesunate has recently completed phase III trials in humans. The safety and efficacy of this compound were shown in four randomized clinical trials in adults and children in Africa and Asia [2–5]. Pyronaridine-artesunate showed better efficacy compared with mefloquine-artesunate for the treatment of uncomplicated falciparum malaria in Cambodia and a non-inferior efficacy compared with artemether-lumefantrine in Africa and Southeast Asia. The emergence of *P. falciparum* resistance to artemisinin and artemisinin derivatives has recently developed in Southeast Asia, manifesting as delayed parasite clearance following treatment with artesunate monotherapy or ACT [6, 7]. Resistance has still developed with the most recent ACT in the form of dihydroartemisinin-piperaquine, which demonstrated less than 70 % efficacy [8, 9]. In areas where the resistance of artemisinin is emerging, partner drugs are under increasing pressure for the selection of resistance, and new therapeutics are limited. Thus, it is important to use an ACT in which its partner drug shows a different mode of action or mechanism of resistance. The in vitro responses to pyronaridine and piperaquine were differently distributed in a triple normal distribution model for pyronaridine and a quadruple normal distribution model for piperaquine [10]. Significant positive in vitro cross-susceptibility was observed between pyronaridine and piperaquine (coefficient of determination of 0.20–0.23) [11, 12]. In vitro and ex vivo responses to piperaquine were not associated with the K76T mutation in the *P. falciparum* chloroquine resistance transporter gene (*pfcrt*) [13, 14]. Few data are available on the association between pyronaridine susceptibility and polymorphisms in the genes involved in antimalarial drug resistance. A study using 23 *P. falciparum* strains showed that there was no significant association between in vitro responses to pyronaridine and *pfcrt* polymorphism [15]. The objective of the present study was to investigate the association between *ex vivo* responses to pyronaridine and the K76T mutation in the *pfcrt* gene in 296 *P. falciparum* African isolates.

In total, 296 *P. falciparum* isolates were collected between April 2008 and August 2012 from patients hospitalized in France with imported malaria from African malaria-endemic countries (Angola, Benin, Burkina Faso, Cameroon, Central African Republic, Chad, Comoros, Congo, Ivory Coast, Gabon, Gambia, Ghana, Guinea, Madagascar, Mali, Mauritania, Mozambique, Niger, Senegal, Togo, and Zambia). Informed consent was not required for this study because the sampling and testing procedures were conducted according to the French national recommendations for the care and surveillance of malaria. The *ex vivo* responses to pyronaridine (Shin Poong Pharm Co., Seoul, Korea) and chloroquine (St. Louis, MO, USA) (control for *pfcrt* polymorphism) were assessed as previously described using a standard 42-h 3H-hypoxanthine uptake inhibition method [10]. Batches of plates were tested and validated using the chloroquine-susceptible 3D7 strain (West Africa) and the chloroquine-resistant W2 strain (Indochina) (MR4, Virginia, USA) in three to six independent experiments. Nucleic acid extraction and *pfcrt* single-nucleotide polymorphism identification were previously described [14].

The pyronaridine IC_50_ values (inhibitory concentration 50 %) ranged from 0.55 to 80.0 nM (Fig. 1). The geometric mean was 20.8 ± 14.6 nM (standard deviation). *Ex vivo* responses to pyronaridine were significantly associated with the K76T mutation (*p*-value = 0.020), and similar results were obtained for chloroquine IC_50_ (*p*-value < 0.001). Sixteen isolates (5.4 %) had an IC_50_ greater than 60 nM and were considered to display reduced susceptibility to pyronaridine in vitro [10]. The reduced susceptibility to pyronaridine, defined as IC_50_ > 60 nM, was significantly associated with the K76T mutation (*p*-value = 0.004). The odds ratio for reduced susceptibility to pyronaridine associated with the K76T mutation was 4.47 (95 % CI [1.39–18.84]). The in vitro resistance to chloroquine, defined as IC_50_ > 100 nM, was also significantly associated with the K76T mutation (*p*-value < 0.001). The odds ratio for reduced susceptibility to chloroquine associated with the K76T mutation was 96.4 (95 % CI [41.8–244.8]). Using Bayesian mixture modelling, the 296 pyronaridine IC_50_values were classified into three components: component A (IC_50_ median 15.9 nM), component B (IC_50_ median 34.2 nM) and component C (IC_50_ median 63.3 nM) (Table 1). The pyronaridine medians were significantly different in the three components (Kruskal-Wallis test, *p*-value < 0.001). The proportion of isolates in each group was 59.8 % for component A, 30.1 % for component B and 10.1 % for component C. The K76T mutation represented 46.3 % of the isolates in component A, 47.2 % of the isolates in component B and 73.3 % of the isolates in component C (Kruskal-Wallis test, *p*-value = 0.021).

**Fig. 1:**
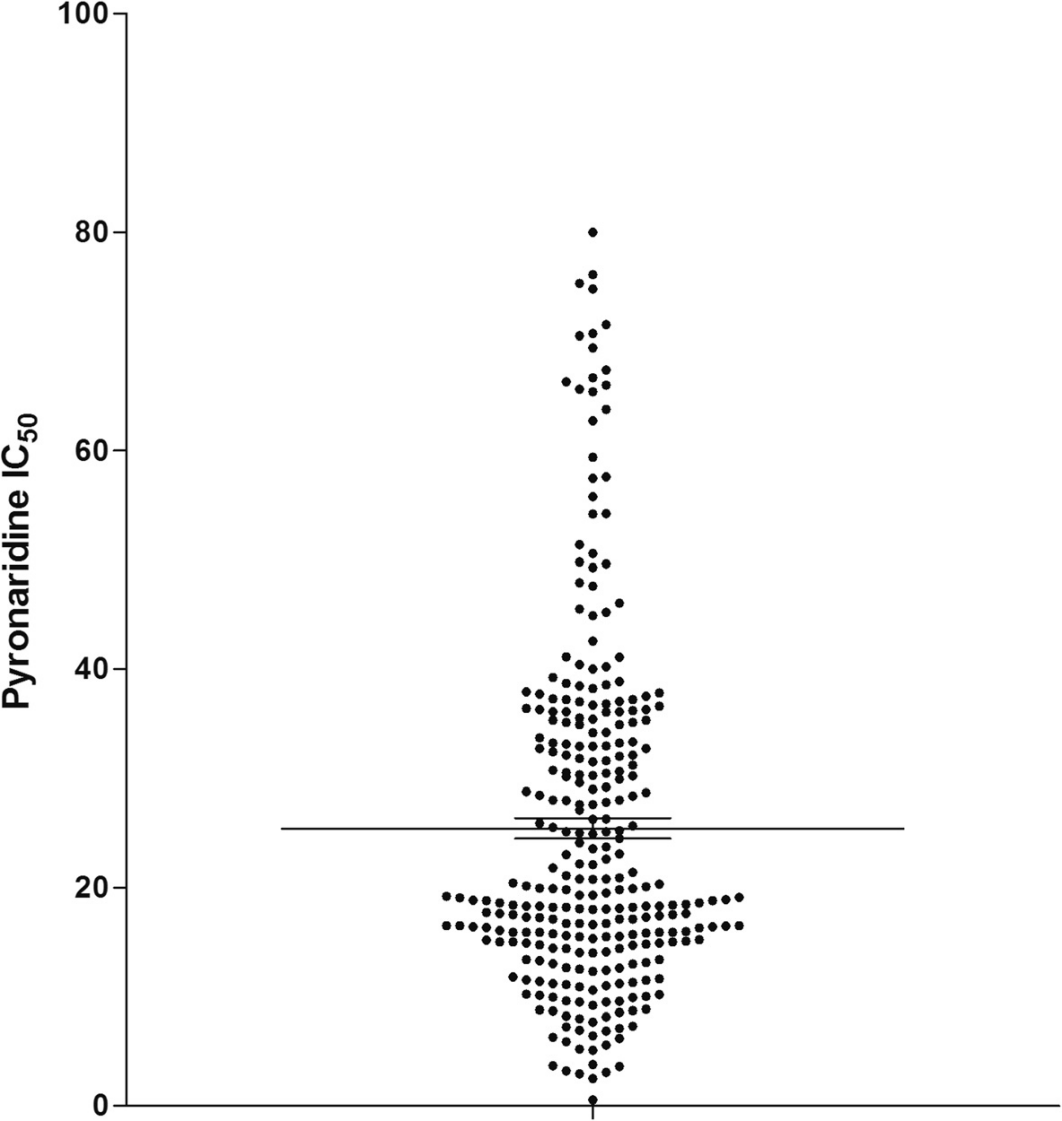
Pyronaridine median and 25 and 75 percentiles of the 50 % inhibitory concentration (IC50 in nM) of 296 African *Plasmodium falciparum* isolates

**Table 1:** Distribution of the IC_50_ of pyronaridine and chloroquine and the K76T mutation according to the three components defined by the Bayesian mixture modeling approach

	Component A	Component B	Component C	*P*-value
No of isolates	177	89	30	
Pyronaridine median IC_50_	15.9 nM	34.2 nM	63.3 nM	<0.001
Pyronaridine 25 % percentile	11.2 nM	30.4 nM	51.2 nM	
Pyronaridine 75 % percentile	18.5 nM	37.1 nM	69.7 nM	
Chloroquine median IC_50_	57.0 nM	90.6 nM	240.5 nM	0.003
Chloroquine 25 % percentile	18.3 nM	19.7 nM	71.2 nM	
Chloroquine 75 % percentile	207 nM	273 nM	301 nM	
No of K76T mutation (%)	82 (46.3)	42 (47.2)	22 (73.3)	0.021

The results of the present study showed that *ex vivo* reduced susceptibility to pyronaridine, i.e., IC_50_ > 60 nM, was associated with the K76T mutation in the *pfcrt* gene. These *ex vivo* results are in contrast with the in vitro results in 23 *P. falciparum* strains in which the in vitro to pyronaridine were not associated with *pfcrt* polymorphisms [15]. However, none of the 23 strains showed reduced susceptibility to pyronaridine. In 59 field isolates from Kenya, pyronaridine was more active in vitro against parasites harbouring the wild-type sequence than against those harbouring the K76T mutation (IC_50_ of 6 versus 20 nM). However, this difference was not significant (*p*-value > 0.05) [16]. The odds ratio for the K76T mutation associated with reduced susceptibility to pyronaridine was 4.47, whereas the odds ratio for in vitro resistance to chloroquine was 96.4. The resistance to pyronaridine is certainly multigenic, and additional polymorphisms in other genes could also be involved in this resistance.

In contrast with piperaquine, in which its in vitro responses are not associated with *pfcrt* polymorphism in isolates from Africa and Asia [13, 14, 17, 18] but rather associated with repeat polymorphisms in an ABC transporter gene, *pfmdr6* [19], pyronaridine mechanisms of resistance are different than those involved in piperaquine. Pyronaridine-artesunate could be used in areas where resistance to other ACTs has already emerged.

Pyronaridine-artesunate successfully treats artemisinin-resistant *P. berghei* parasites, while artemether-lumefantrine, artesunate-amodiaquine, artesunate-mefloquine and dihydroartemisinin-piperaquine are not effective [20]. Pyronaridine-artesunate showed better efficacy than mefloquine-artesunate for the treatment of uncomplicated falciparum malaria in Cambodia and a non-inferior efficacy compared with that of artemether-lumefantrine in Africa and Southeast Asia [2–4]. Pyronaridine-artesunate showed greater than 95 % efficacy when used as an initial falciparum malaria treatment versus the re-treatment of subsequent episodes in a multi-site trial in Mali, Burkina Faso and Guinea [21]. In addition, pyronaridine-artesunate is also affective in the treatment of acute uncomplicated *P. vivax* malaria [5]. Pyronaridine-artesunate is an alternative artemisinin-based combination treatment for malaria in sub-Saharan Africa.

## Ethical approval

According to the French legislation, bio-banking and secondary use for scientific purposes of human clinical samples are possible as long as the corresponding patients are informed and have not indicated any objections. This requirement was fulfilled here since information is given to every patient through a hospital notice entitled “Information for Patients,” and no immediate or delayed patient opposition was reported by the hospital clinicians to the French Malaria Reference Center. Moreover, samples received at the French Malaria Reference Center were registered and declared for research purposes as a bio-bank for the French National Institute of Health Survey. No institutional review board approval is required according to French legislation (article L. 1111–7 du Code de la Santé Publique, article L. 1211–2 du Code de Santé Publique, articles 39 et suivants de la loi 78–17 du 6 janvier 1978 modifiée en 2004 relative à l’informatique, aux fichiers, et aux libertés).

## Abbreviations

ACT: artemisinin-based combination therapy
IC_50_: inhibitory concentration 50 %
*pfcrt*: *P. falciparum* chloroquine resistance transporter gene
WHO: World Health Organization

## References

[1] Croft SL, Duparc S, Arbe-Barnes SJ, Craft JC, Shin CS, Fleckenstein L (2012). Review of pyronaridineanti-malarial properties and product characteristics. Malar J.

[2] Tshefu AK, Gaye O, Kayentao K, Thompson R, Bhatt KM, Sesay SS (2010). Efficacy and safety of a fixed-dose oral combination of pyronaridine-artesunate compared with artemether-lumefantrine in children and adults with uncomplicated *Plasmodium falciparum* malaria: a randomised non-inferiority trial. Lancet.

[3] Kayentao K, Doumbo OK, Pénali LK, Offianan AT, Bhatt KM, Kimani J (2012). Pyronaridine-artesunate granules versus artemether-lumefantrine crushed tablets in children with *Plasmodium falciparum* malaria: a randomized controlled trial. Malar J.

[4] Rueangweerayu R, Phyo AP, Uthaisin C, Poravuth Y, Binh TQ, Tinto H (2012). Pyronaridine–artesunate versus mefloquine plus artesunate for malaria. N Engl J Med.

[5] Poravuth Y, Socheat D, Rueangweerayut R, Uthaisin C, Pyae Phyo A, Valecha N (2011). Pyronaridine-artesunate versus chloroquine in patients with acute Plasmodium vivax malaria: a randomized, double-blind, non-inferiority trial. PLoS One.

[6] Dondorp AM, Nosten F, Yi P, Das D, Phyo AP, Tarning J (2009). Artemisinin resistance in *Plasmodium falciparum* malaria. N Engl J Med.

[7] Ashley EA, Dhorda M, Fairhurst RM, Amaratunga C, Lim P, Suon S (2015). Spread of artemisinin resistance in *Plasmodium falciparum* malaria. N Engl J Med.

[8] Leang R, Taylor WRJ, MeyBouth D, Song L, Tarning J, Chuor Char M (2015). Evidence of *Plasmodium falciparum* malaria multidrug resistance to artemisinin and piperaquine in Western Cambodia: Dihydroartemisinin-piperaquine open-label multicentre clinical assessment. Antimicrob Agents Chemother.

[9] Spring M, Lin JT, Manning JE, Vanachayangkul P, Somethy S, Bun R (2015). Dihydroartemisinin-piperaquine failure associated with a triple mutant including Kelch13 C580Y in Cambodia: an observational cohort study. Lancet Infect Dis.

[10] Pascual A, Madamet M, Briolant S, Gaillard T, Amalvict R, Benoit N (2015). Multinormalin vitro distribution of *Plasmodium falciparum* susceptibility to piperaquine and pyronaridine. Malar J.

[11] Pascual A, Parola P, Benoit-Vical F, Simon F, Malvy D, Picot S (2012). Ex vivo activity of the ACT new components pyronaridine and piperaquine in comparison with conventional ACT drugs against isolates of *Plasmodium falciparum*. Malar J.

[12] Price RN, Marfurt J, Chalfein F, Kenagalem E, Piera KA, Tjitra E (2010). In vitro activity of pyronaridine against multidrug-resistant *Plasmodium falciparum*and *Plasmodium vivax*. Antimicrob Agents Chemother.

[13] Briolant S, Henry M, Oeuvray C, Amalvict R, Baret E, Didillon E (2010). Absence of association between piperaquine in vitro responses and polymorphisms in the *pfcrt*, *pfmdr1*, *pfmrp*, and *pfnhe* genes in *Plasmodium falciparum*. Antimicrob Agents Chemother.

[14] Pascual A, Madamet M, Bertaux L, Amalvict R, Benoit N, Travers D (2013). *In vitro*piperaquine susceptibility is not associated with the *Plasmodium falciparum* chloroquine resistance transporter gene. Malar J.

[15] Pradines B, Briolant S, Henry M, Oeuvray C, Baret E, Amalvict R (2010). Absence of association between pyronaridine*in vitro* responses and polymorphisms involved in quinoline resistance in *Plasmodium falciparum*. Malar J.

[16] Okombo J, Kiara SM, Mwai L, Pole L, Ohuma E, Ochola LI (2012). Baseline *In vitro* activities of the antimalarialspyronaridine and methylene blue against *Plasmodium falciparum* isolates from Kenya. Antimicrob Agents Chemother.

[17] Mwai L, Kiara SM, Abdirahman A, Pole L, Rippert A, Diriye A (2009). In vitro activities of piperaquine, lumefantrine, and dihydroartemisinin in Kenyan *Plasmodium falciparum* isolates and polymorphisms in *pfcrt* and *pfmdr1*. Antimicrob Agents Chemother.

[18] Hao M, Jia D, Li Q, He Y, Yuan L, Xu S (2013). *In vitro* sensitivities of *Plasmodium falciparum* isolates from the China-Myanmar border to Piperaquine and association with polymorphisms in candidate genes. Antimicrob Agents Chemother.

[19] Okombo J, Abdi AI, Kiara SM, Mwai L, Pole L, Sutherland CJ (2013). Repeat polymorphisms in the low-complexity regions of *Plasmodium falciparum* ABC transporters and associations with in vitro antimalarial responses. Antimicrob Agents Chemother.

[20] Henrich PP, O'Brien C, Sáenz FE, Cremers S, Kyle DE, Fidock DA (2014). Evidence for pyronaridine as a highly effective partner drug for treatment of artemisinin-resistant malaria in a rodent model. Antimicrob Agents Chemother.

[21] Sagara I, Beavogui AH, Zongo I, Soulama I, Borghini-Fuhrer I, Fofana B (2015). Safety and efficacy of re-treatments with pyronaridine-artesunate in African patients with malaria: a substudy of the WANECAM randomised trial. Lancet Infect Dis.

